# Supplementary figures and images for: Replication of the Salmonella Genomic Island 1 (SGI1) triggered by helper IncC conjugative plasmids promotes incompatibility and plasmid loss
Source: PLoS Genet. 2020 Aug 6;16(8):e1008965. doi: 10.1371/journal.pgen.1008965 (PMC7433901; doi:10.1371/journal.pgen.1008965)

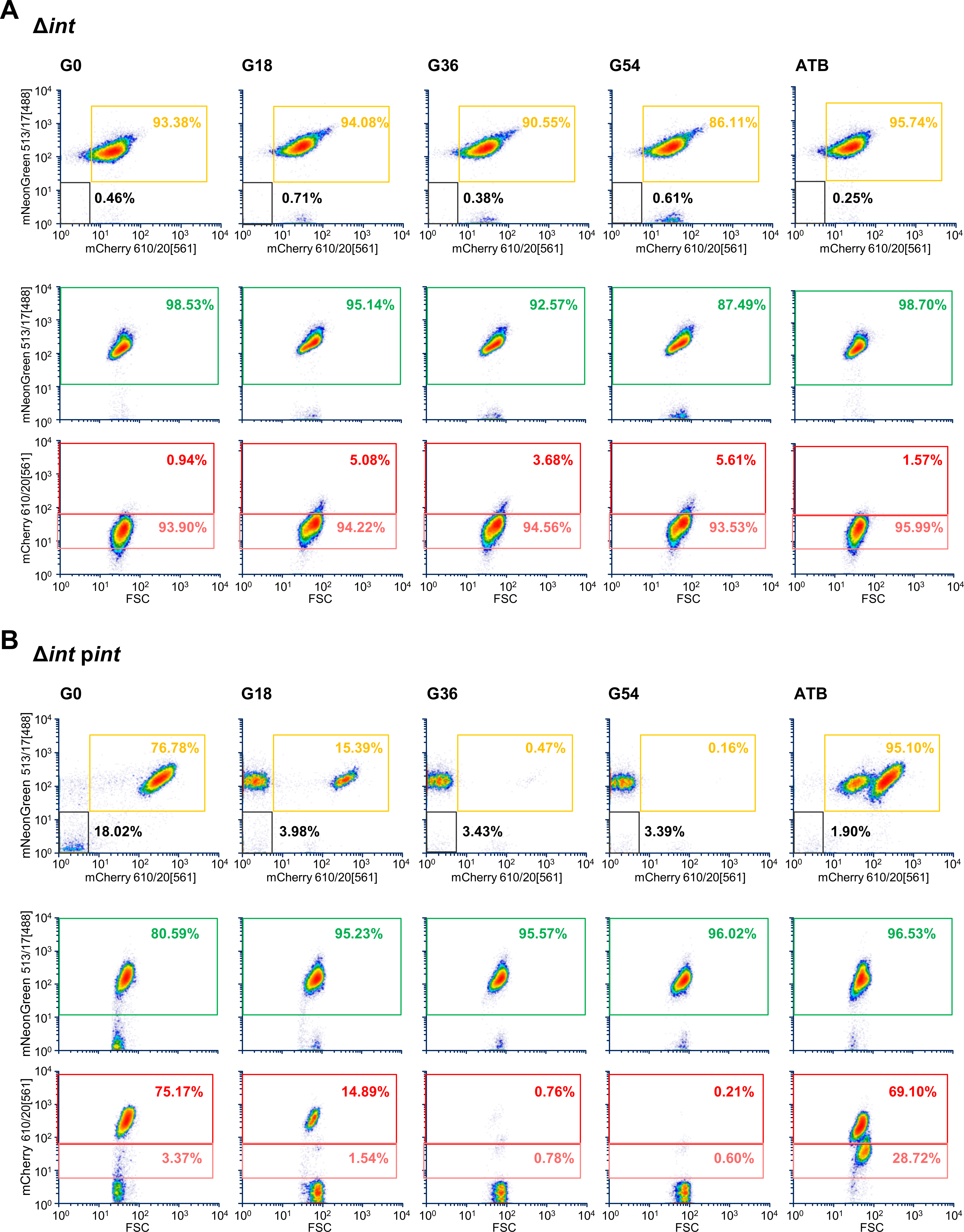

Supplement: S1 Fig — (A) Evolution of the percentage of E. coli KH95 cells bearing pVCR94Green and SGI1Red Δint over 54 generations in the absence of antibiotics as monitored using FC. (B) Complementation of SGI1Red Δint with pint. KH95 carried pVCR94GreenSp in these assays. (TIF) [file pgen.1008965.s001.tif]

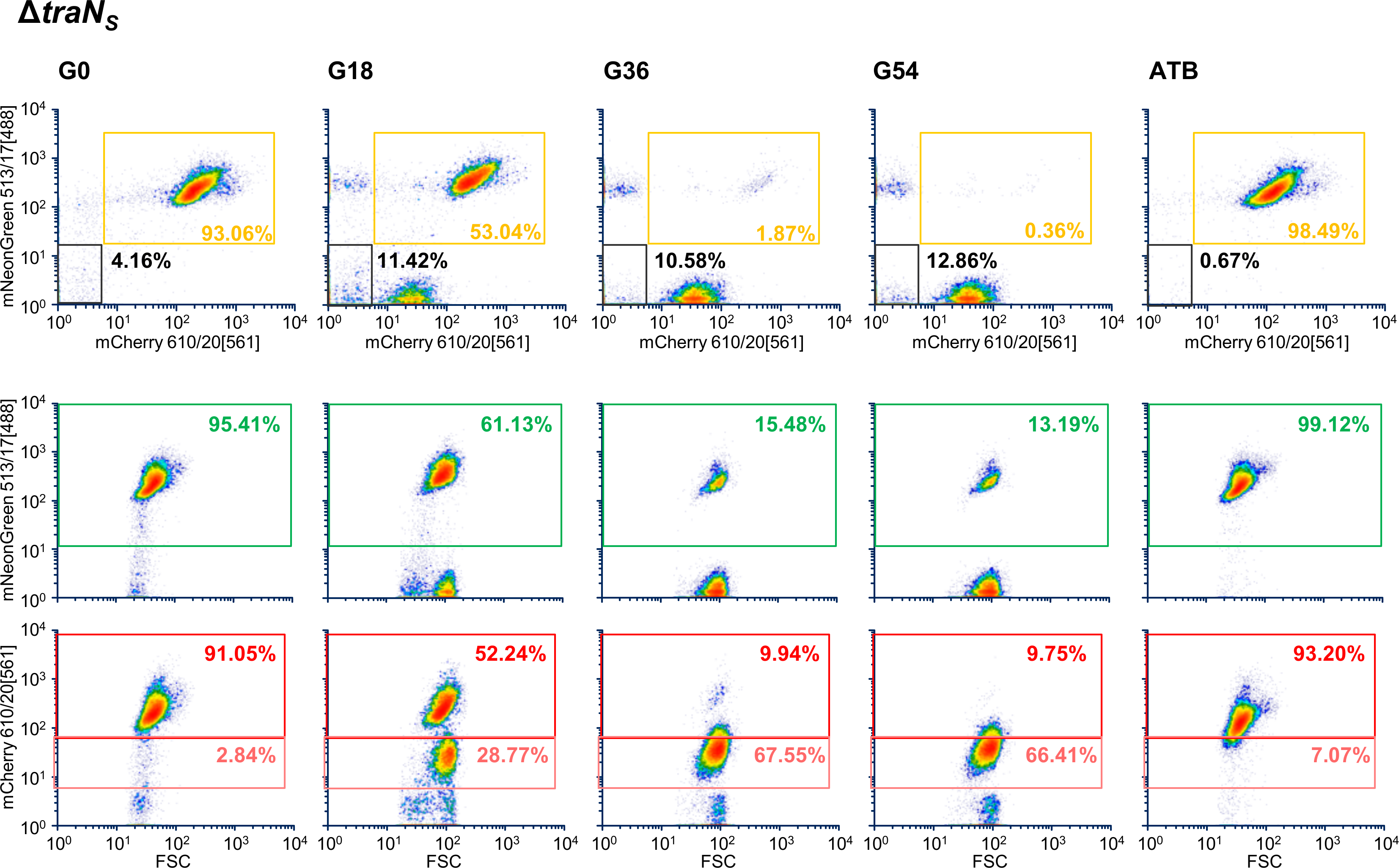

Supplement: S2 Fig — Evolution of the percentage of E. coli KH95 cells bearing SGI1Red ΔtraNS and pVCR94Green over 54 generations in the absence of antibiotics as monitored using FC. (TIF) [file pgen.1008965.s002.tif]

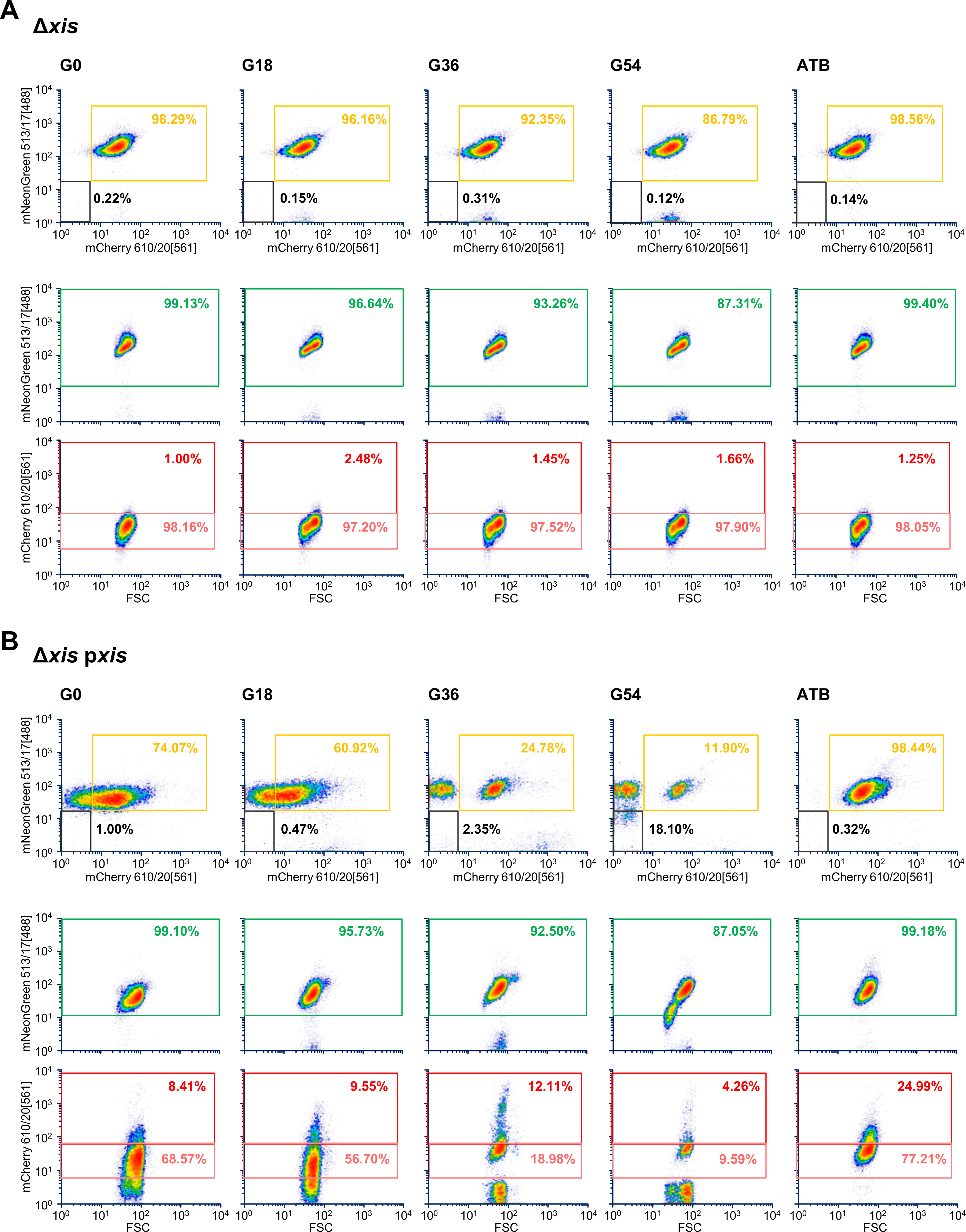

Supplement: S3 Fig — (A) Evolution of the percentage of E. coli KH95 cells bearing pVCR94Green and SGI1Red Δxis over 54 generations in the absence of antibiotics as monitored using FC. (B) Complementation of SGI1Red Δxis with pxis. KH95 carried pVCR94GreenSp in these assays. (TIF) [file pgen.1008965.s003.tif]

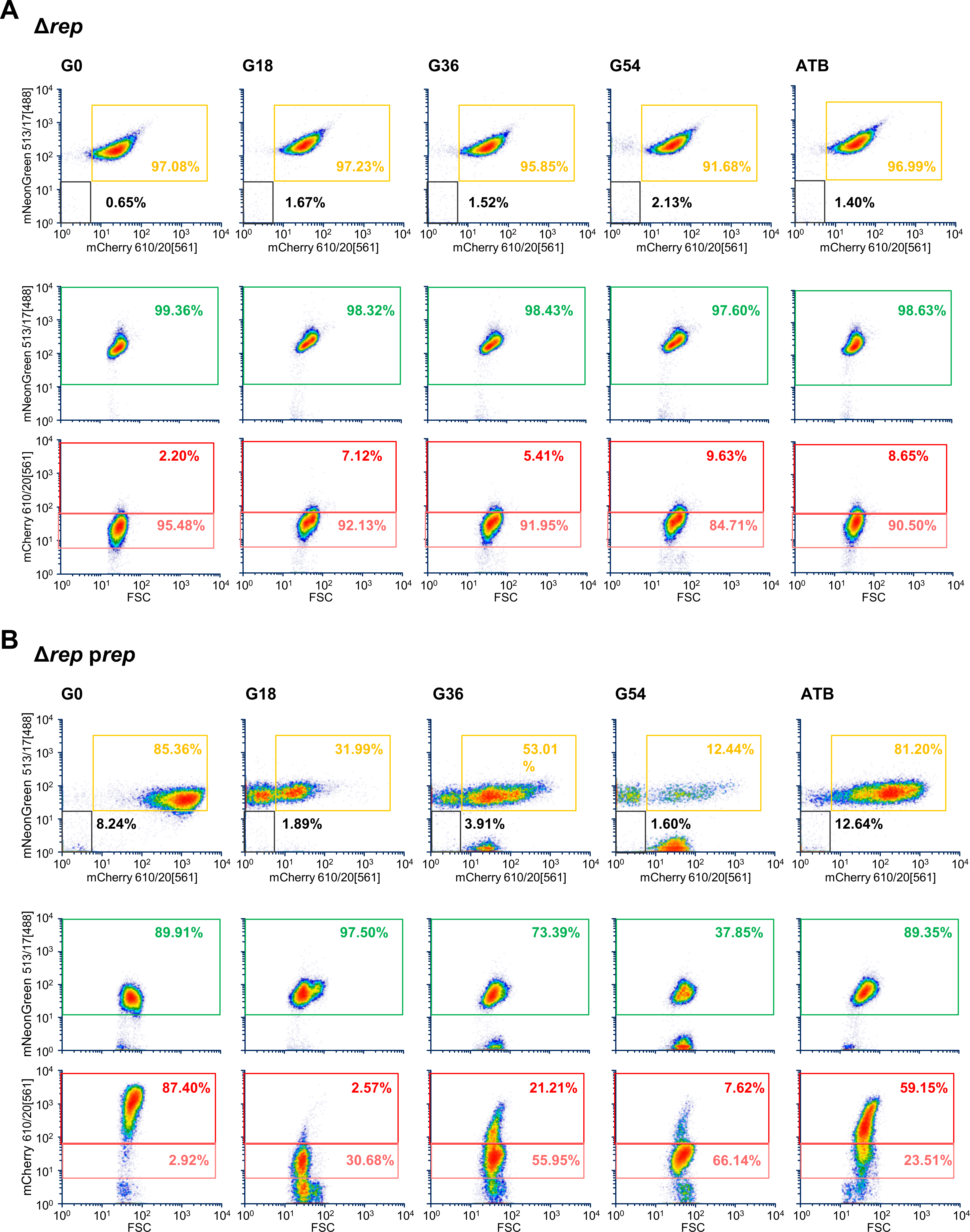

Supplement: S4 Fig — (A) Evolution of the percentage of E. coli KH95 cells bearing pVCR94Green and SGI1Red Δrep over 54 generations in the absence of antibiotics as monitored using FC. (B) Complementation of SGI1Red Δrep with prep. KH95 carried pVCR94GreenSp in these assays. (TIF) [file pgen.1008965.s004.tif]

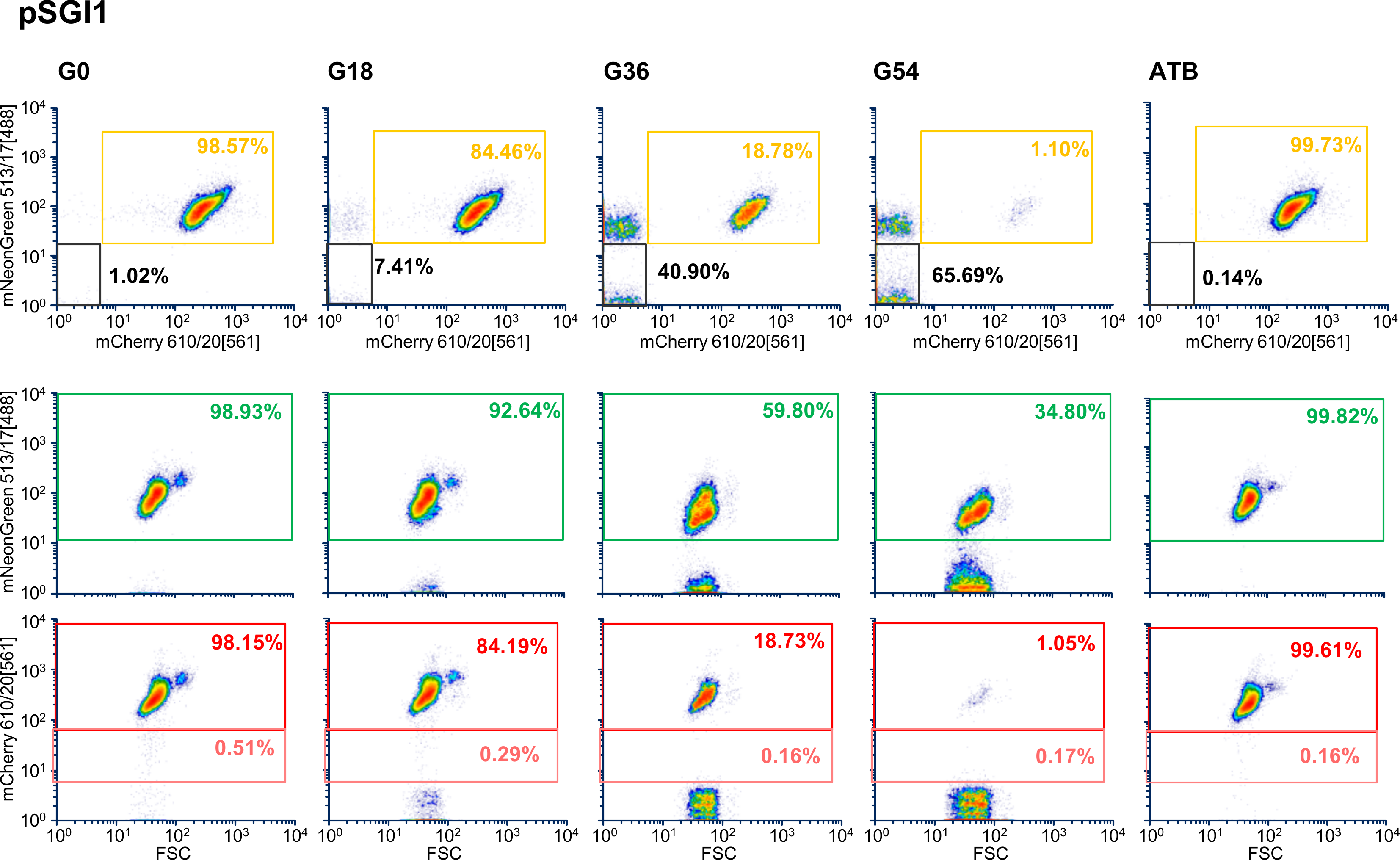

Supplement: S5 Fig — (TIF) [file pgen.1008965.s005.tif]

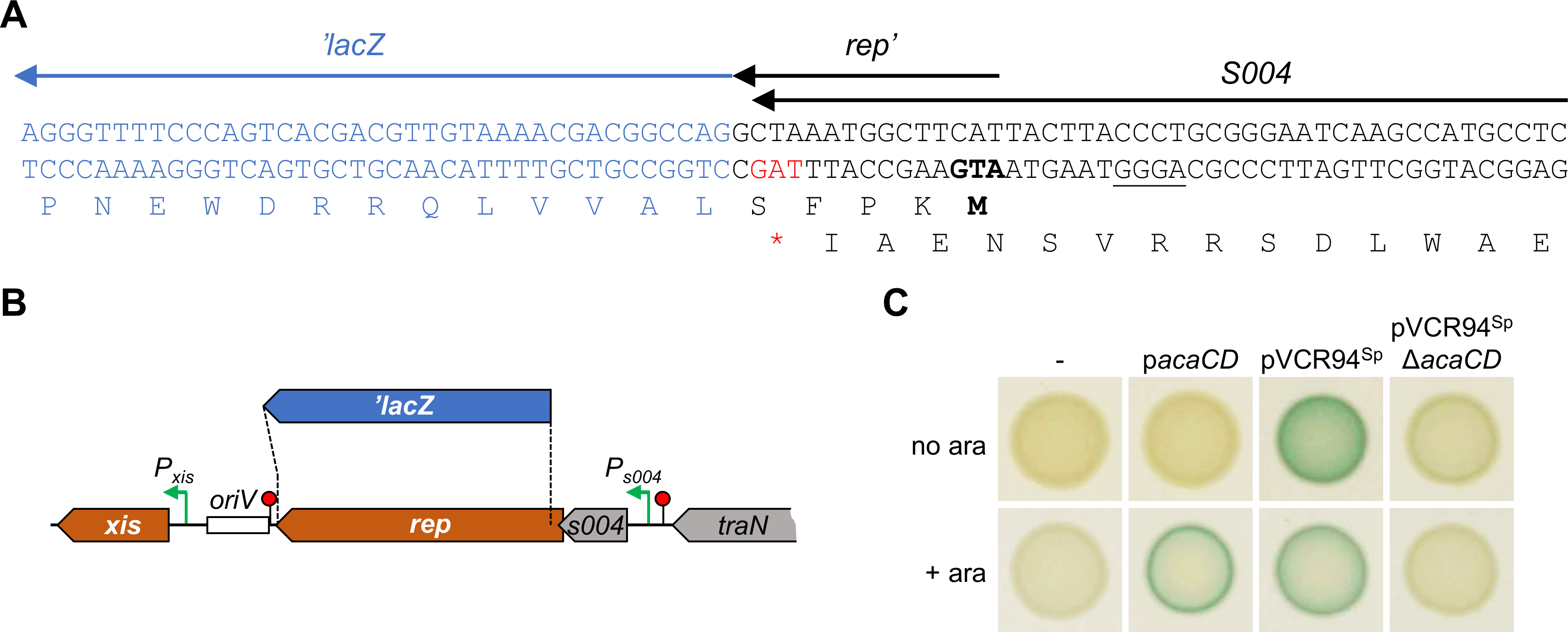

Supplement: S6 Fig — (A) Sequence of the rep’-’lacZ translational fusion. The open reading frames are indicated by arrows. The predicted Shine-Dalgarno sequence of rep is underlined and its start codon is shown in bold. The red asterisk indicates the stop codon (in red) of S004. The sequence of lacZ is shown in blue whereas the sequence of SGI1 is shown in black. Predicted translation product are shown below the nucleotide sequence. (B) Schematic representation of the rep’-’lacZ translational fusion in SGI1. (C) β-galactosidase assays of the translational rep’-’lacZ fusion in SGI1Kn performed in IncC-free cells (-), and in the presence of pVCR94Sp, its ΔacaCD mutant or pacaCD without or with arabinose (+ara). (TIF) [file pgen.1008965.s006.tif]

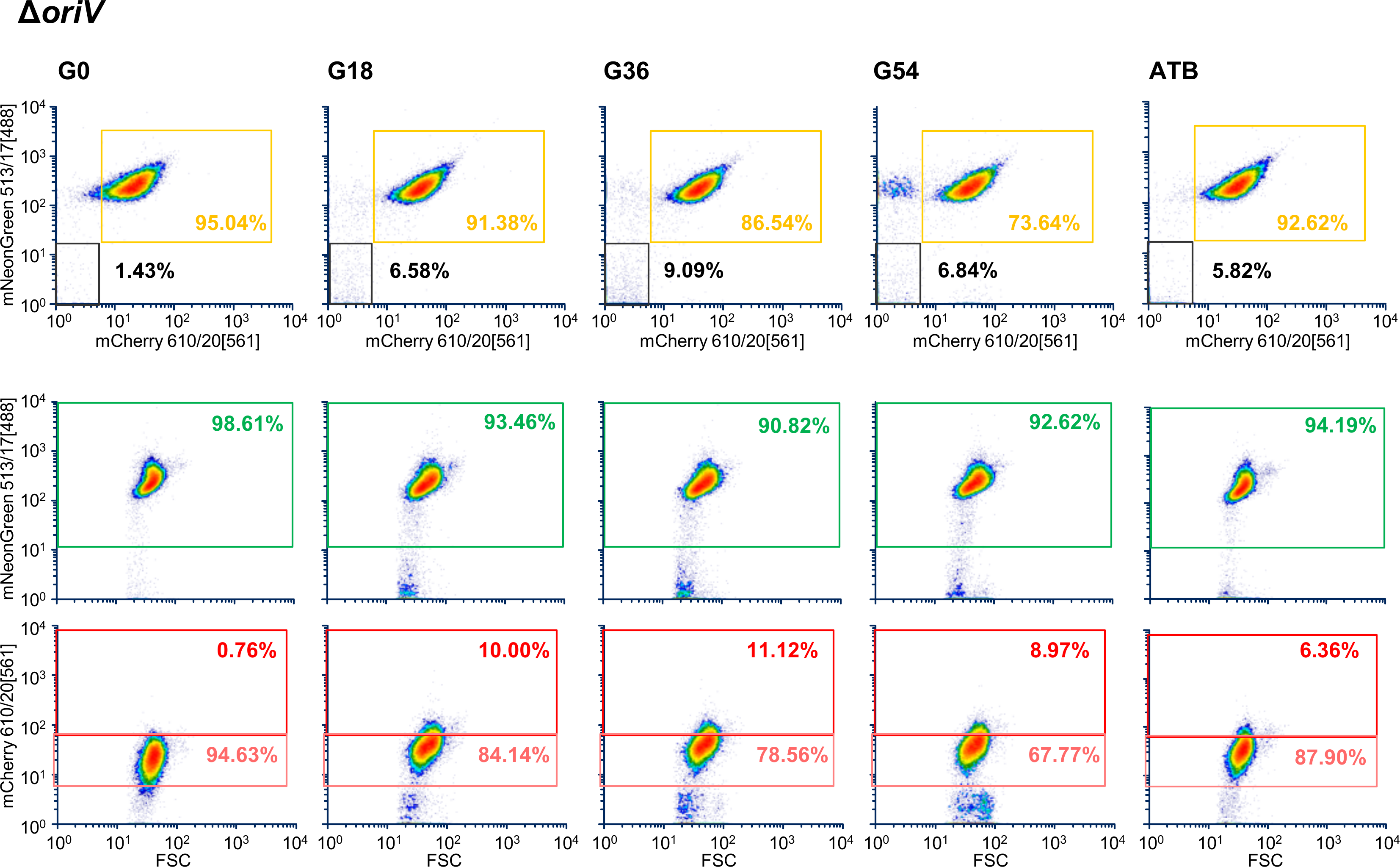

Supplement: S7 Fig — Evolution of the percentage of E. coli KH95 cells bearing pVCR94Green and SGI1Red ΔoriV over 54 generations in the absence of antibiotics as monitored using FC. (TIF) [file pgen.1008965.s007.tif]
