# Supplementary material for: Replication of the Salmonella Genomic Island 1 (SGI1) triggered by helper IncC conjugative plasmids promotes incompatibility and plasmid loss
Source: PLoS Genet. 2020 Aug 6;16(8):e1008965. doi: 10.1371/journal.pgen.1008965 (PMC7433901; doi:10.1371/journal.pgen.1008965)
Supplement: S1 Table. Primers used in this study — (DOCX) [file pgen.1008965.s008.docx]

**S1 Table.** Primers used in this study.

| **Primer** | **Nucleotide sequence (5’ to 3’)*^a^*** |  |
| --- | --- | --- |
| Fw-EcoRI-RBSNG | TCAGGAATTCAAAGAGGAGAAATACTAGATGGTTTCTAAAGGAGAAGA | Cloning of *mNeongreen* in pBAD30 |
| Rv-NG-KpnI | CTGCAGGTACCTTATTTATATAATTCATCCA |  |
| Fw-EcoRI-RBSMC | TCAGGAATTCAAAGAGGAGAAATACTAGATGGTGAGCAAGGGCGAGGA | Cloning of *mCherry* in pBAD30 |
| Rv-MC-KpnI | CTGCAGGTACCCTACTTGTACAGCTCGTCCA |  |
| FwpBADInsMCSGI1 | CCGGAAGCTTCTATGGATTGAATAAAAACAAAGGCTAATGGACGAAGCAGGGATTC | Insertion of *mCherry* into SGI1 |
| RvpBADInsMCSGI1 | ATAGGCAGTTAACGTTAAGTCTATAATGAATAAAAATCATGAGCGGATACATATTTGA |  |
| FwpBADInsNGpVCR | AGGGGGTACAACATAGCAAATCAAAGGTTCTAAAAGAATGGACGAAGCAGGGATTC | Insertion of *mNeongreen* into pVCR94 |
| RvpBADInsNGpVCR | CGCTTCCATATTGGAGCTGGAGACGAATAAGGGGGCTCATGAGCGGATACATATTTGA |  |
| SGI1In104cm2.f | TATTTTAACATGTATATAGATTAAATTCCAATCAACTTGTCGGAATAGGAACTTCATTTA | Deletion of In104 |
| SGI1In104cm2.r | CAAACCACCATTTTCTTAACAGTTCATAACTTAGCAAGTTGCCTACCTGTGACGGAAGAT |  |
| SGI1delint.for | AGTTAAAATTTTGGAGTAACGGGAGCAAAGCGGAGCAATTGTGTAGGCTGGAGCTGCTTCG | Deletion of *int* (*S001*) |
| SGI1delint.rev | ACATCACGAGTTTTCCTCGCGGCCGTAGGGCATCTTAAAGTTATTACATATGAATATCCTCCTTA |  |
| SGI1delxis.for | AACTAGGTCTATCAATAACTGGTGTGTAACGGGTAGGCTGGTGTAGGCTGGAGCTGCTTCG | Deletion of *xis* (*S002*) |
| SGI1delxis.rev | CTCACTGCGGAAAGTCCGTATTGAATAAAACACTTTAAGATTATTACATATGAATATCCTCCTTA |  |
| SGI1deIRep.for | ATGGCTTGATTCCCGCAGGGTAAGTAATGAAGCCATTTAGCTAATAAGTGTAGGCTGGAGCTGCTTCG | Deletion of *rep* (*S003*) |
| SGI1deIRep.rev | CATAAAAAAACCGGCACATTGGCCGGTTATTAAGTCATTACATATGAATATCCTCCTTA |  |
| SGI1dels005.for | CGACATAGCATTATCCAAACTAAAAAGCTGGAGAAATGCTGTGTAGGCTGGAGCTGCTTCG | Deletion of *traN_S_* (*S005*) |
| SGI1dels005.rev | TTGATTTACTGGAGAAATTTTGAGGGTGAACCCCCGTAAACATATGAATATCCTCCTTA |  |
| Fw-DelOriRSGI1 | TTAATAACCGGCCAATGTGCCGGTTTTTTTATGTTATTGTGTAGGCTGGAGCTGCTTCG | Deletion of *oriV* |
| Rv-DelOriRSGI1 | TATTAACGCCTGAACATGGTTCAACTATAGGTATCTTTCATATGAATATCCTCCTTA |  |
| rep-lacZ.f | TTGATTCCCGCAGGGTAAGTAATGAAGCCATTTAGCCTGGCCGTCGTTTTACAACGTCG | *rep*’-’*lacZ* fusion |
| rep-lacZ.r2 | ATAAAAAAACCGGCACATTGGCCGGTTATTAAGTCAGCAGCATTACACGTCTTGAG | *rep*’-’*lacZ* fusion |
| rep-lacZdelxis.r2 | CTCACTGCGGAAAGTCCGTATTGAATAAAACACTTTAAGAGCAGCATTACACGTCTTGAG | *rep*’-’*lacZ* fusion and *xis* deletion |
| SGI1intEcoRI.for | GAATTCAAGGAGGAATAATAAATGAAGGTATCAGTAAACAAGCGTA | Cloning of *int* in pBAD30 |
| SGI1intEcoRI.rev | GAATTCTTAAAGTGTTTTATTCAATACGGACTT |  |
| SGI1xisEcoRIb.for | GAATTCAAGGAGGAATAATAAA | Cloning of *xis* in pBAD30 |
| SGI1xisEcoRI.rev | GAATTCTTAAGATGCCCTACGG |  |
| Fw-KpnI-Rep | GGTACCAAAGAGGAGAAATACTAGATGAAGCCATTTAGCCAACT | Cloning *rep* of in pBAD30 |
| Rv-Rep-SalI | GTCGACTCAATTACGGTATGGAATCG |  |
| qattBFw | AACATCTACAACAGGGCAAAG | qPCR *attB* |
| qattBRv | GAGGAATAACAGGAGTGGTAAC | qPCR *attB* |
| qattPFw | CGGATGAAGATCAGGGAGAA | qPCR *attP* |
| qattPRv | GGTTGTTTTGATATGTTTTGAAAGG | qPCR *attP* |
| qS026Fw | TGTCATCAGAAAGAACAAGCTC | qPCR *S026* |
| qS026Rv | GCGTTTTTATTCTGTTGCCC | qPCR *S026* |
| qFwlfjct | GGAATTTACTTCAGACGACCT | qPCR SGI1 left junction |
| qRvlfjct | GGAGCAAAATCGTGAGAAGG | qPCR SGI1 left junction |
| qFwpVCR | AAGAGAACCAAAGACAAAGACC | qPCR pVCR94 |
| qRvpVCR | CACCTTCACCGTGAAATGC | qPCR pVCR94 |
| qdnaBFw | ACGATTTTTACACCCGCCCAC | qPCR *dnaB* |
| qdnaBRv | ATCATCTCACGGACAACGGCAC | qPCR *dnaB* |
| qhicBFw | GCTTATCCCTTTACCTTCGCC | qPCR *hicB* |
| qhicBRv | TAACTCTTTGCCAAGCGCC | qPCR *hicB* |
| qthdfFw | GATAATGACACTATCGTAGCCC | qPCR *trmE* |
| qthdfRv | GCAGTTCCAGCACATCTTC | qPCR *trmE* |
| rep_rt-R | CCAGTCACTTTCTTAACTTTTG | Reverse transcription of *rep* |
| rep_1F | TTGGCGTTATCTGCTTGTAG | Amplification of *rep* |
| rep_1R | CAAGAGCACTGCTATTAAGCTG | Amplification of *rep* |
| s004_F | ATGCTGGAGAAATTTATGCAG | Amplification of *s004* |
| s004_R | GCCTCCAACTGATCTAACCTG | Amplification of *s004* |
| traN_F | GCATATCAAGTTCTCATGGATG | Amplification of *traN_S_* |
| traN_R | CTTTGATCTACAACACGCTG | Amplification of *traN_S_* |

*^a^* Cloning sites are underlined
