# Supplementary material for: Replication of the Salmonella Genomic Island 1 (SGI1) triggered by helper IncC conjugative plasmids promotes incompatibility and plasmid loss
Source: PLoS Genet. 2020 Aug 6;16(8):e1008965. doi: 10.1371/journal.pgen.1008965 (PMC7433901; doi:10.1371/journal.pgen.1008965)
Supplement: Attachment — Submitted filename: Response to reviewers.docx [file pgen.1008965.s009.docx]

**Below the responses to reviewers are indicated in blue. Line numbers in parenthesis refer to the modified manuscript.**

**Please note that a reference to “Data not shown” was removed line 167. Since this reference did not provide any substantial information, we decided to remove it for the sake of simplicity instead of adding an additional supplementary material.**

**Reviewer #1:** Huguet and coworkers study the apparent incompatibilities between the Salmonella genomic island SIG1 and plasmids of the type IncC/IncA. They label SIG1 and plasmid with different inducible fluorescent reporter proteins to quantify presence of both elements in single cells by flow cytometry under different conditions and various mutant backgrounds. This was further backed up by qPCR and other DNA analyses, and finally by proposing similarities to related systems.

The main conclusion of the work is that SIG1 is replicating in its excised state, which is dependent on presence of an IncC-type plasmid with a transcription activator that controls expression of the SIG1 rep-gene. Yet, this replication causes an incompatibility, which leads to preferential loss of the plasmid.

COMMENTS

1) I have very few criticisms on this work. It is very complete and clearly written. The results are convincing. There are clear differences in the expression levels of the fluorescence markers, which allows the authors to conclude that must correspond to three SIG1 states: a chromosomally integrated state (low fluo); an excised state (intermediate fluo) and a replicated excised state (high fluorescence). The plasmid leads to two fluorescent states: presence (high) or absence (low). This was benchmarked by qPCRs, by isolation of the physical SIG1 form, by different mutations that block the SIG1 from excision or from replicating.

The causal link of SIG1 replication after excision to induction of the Rep factor on SIG1 by the IncC plasmid AcaCD activator complex is very clearly demonstrated as well.

We thank the reviewer for his/her very kind remarks.

2) The results of this work therefore clearly advance our understanding of integrated mobilizable elements that rely on other DNA replicons for their transfer. It is extremely curious that an element like SIG1 needs a plasmid for its excision and for its replication, and then hijacks the conjugative system of the plasmid by replacing part by its own conjugation proteins such that its own transfer is preferred over that by the plasmid. Parasites within parasites!
Apart from the molecular intricacies, the fate of SIG1-type elements is very relevant for spread of antimicrobial compound resistance factors, which are often encoded on them.

SGI1 is indeed a fascinating object of study as its genetic and molecular interactions with its helper plasmids are very complex and much remains to be understood.

3) Having said this: there must be some downsides to the SIG1-type strategy. What if there is cross-activation by a chromosomally integrated acaCD-type gene? Would that lead to loss of SIG1 or would it continue to replicate extra-chromosomally? Did the authors ever try such an experiment?

This is a very interesting question. There are currently no other known chromosomally integrated *acaCD*-type genes besides *sgaCD* (aka *flhCD_SGI1_*) encoded by SGI1 itself. Investigation of the role of *sgaCD* is ongoing. We know that sgaCD expression is tightly regulated to prevent auto-excision and replication of SGI1. However, these data are part of a much broader investigation of the role of *sgaCD* in SGI1 lifecycle and are out of scope in this already large manuscript.

The flow cytometry data seem to suggest that there is some escape in the 'incompatibility' conquest between excised replicating SIG1 and IncC. What happens in cells where IncC continues to be present? Are there any mutations inactivating SIG1 or is it simply a matter of chance?
Indeed there seem to be 'incompatibility' escapees and this phenomenon is under investigation. These cells contain the IncC plasmid and likely integrated SGI1. Two hypotheses could explain this phenomenon. Mutations in the plasmid (inactivation of *acaCD*) or SGI1 (loss of replication) could be an explanation. Alternatively, *acaCD* could remain repressed, preventing excision and replication of SGI1. How repression of *acaCD* expression is alleviated to promote conjugative transfer of the IncC plasmid is still poorly understood.

In addition, it should be noted that SGI1 and IncC plasmids have complex interactions but also counteractions. SGI1 destabilizes the helper plasmid, which encodes at least two toxin-antitoxin (TA) systems. SGI1 also encodes its own TA system (*sgiAT*). How these different TA systems interact with one another and how important they are for the stability and incompatibility of both elements is being investigated as well. However, there is little doubt that even in the absence of selective antibiotic pressure, both SGI1 and its helper plasmid fight for their persistence and spread.

4) l. 120 Why are replication-deficient mutants not evolving spontaneously? You mention here and before that no wild-type strains have been found that carry SIG1 and an IncA/C plasmid.

We have not specifically looked for mutations that inactivate SGI1 replication. These mutants probably exist but would have little evolutionary success in a natural context. This is something that will be studied in follow-up experiments.

5) l. 142-144 maybe this is trivial, but how can you select for the presence of both elements that are incompatible in their replication? Will this not automatically lead to appearance of compensatory mutations somewhere?

The presence of both elements was selected with antibiotics specific to SGI1 and for the IncC plasmid. However, this was not a trivial task due to the strong incompatibility. For instance, all frozen stocks were purified and verified for the presence of both elements and FACS experiments were started with populations containing SGI1 and the helper plasmid with over 95%. This is now indicated in the methods section (lines 584-586).

6) l. 230 Bit an awkward notation to write 8.38 x 10-5%. Maybe write both this and 81% in l. 231 as a proportion.

We modified the text accordingly (line 229).

7) l. 241/242: is there no interference at all by inducing all these different elements by arabinose?

There was no interference that we could detect using arabinose induction for multiple target genes in the same strain. In fact, our initial setup was based on constitutive synthetic promoters from the iGEM registry of standard biological parts for the expression of the fluorescent proteins. Unfortunately, expression was so strong during SGI1 replication that we observed reduction of cell growth and poor survival rate.

8) l. 472. Maybe not so uncertain. The big question seems to be what the actual molecular mechanism is that causes SIG1 replication to interfere with that of IncC. It seems to be a very gradual mechanism (i.e., taking more than 50 generations to lead to most of the reduction in a population), which might be due to the continued reliance of SIG1 on IncC? (If a cell would no longer have IncC, the AcaCD would no longer be expressed and SIG1 is out of business). What happens after SIG1 transfer? Is it lost from a 'donor' cell?

In fact, in normal conditions, with WT SGI1, if the IncC plasmid is lost and AcaCD no longer expressed, it is expected that SGI1 reintegrates into the cell as its integrase gene is constitutively expressed and its recombination directionality factor (excisionase) gene is controlled by AcaCD. We believe that the gradual phenotype is due to the expression of TA systems by both the IncC plasmid and SGI1, slowing down the process of incompatibility over several generations. This is an interesting hypothesis and we will delete these TA systems to test incompatibility in their absence in future experiments.

The fate of SGI1 in donor cells after transfer is currently unknown. However, replication could be a mean to retain SGI1 in donor cells. This is now discussed in more detail (lines 471-476).

9) The figures included in the produced PDF are of embarrasing low quality. However, this happened to us recently as well (which was held against us), but we noticed that this is a mistake in the PLoS Genetics conversion process and should absolutely be brought to the attention of the editorial team and improved.

This is unfortunately out of our control. We regularly experience this poor quality in PDF conversions with many journals.

10) Fig. 1. I noticed in this figure and in some others (e.g., Fig. 2C bottom right) that the FSC signal of the mChe-cells is sometimes lower than that of mNeon (panel C). In which growth phase are these cells measured? Do they have a slightly different cell size?

mCherry consistently yielded lower fluorescence intensity than mNeongreen throughout all our assays. FACS assays were made in stationary phase since fluorescence intensity was too low to be detectable in exponential phase likely due to the fast dilution rate of the proteins in growing cells and low expression from the pBAD promoter (now indicated lines 583 and 588). This observation is independent of cell size since the same cells expressed both fluorescent proteins. In fact, mCherry is known to yield a much lower fluorescent intensity than mNeongreen. This property is well documented in a comparative analysis of several fluorescent proteins. See Heppert JK, Dickinson DJ, Pani AM, et al. Comparative assessment of fluorescent proteins for in vivo imaging in an animal model system. *Mol Biol Cell*. 2016;27(22):3385-3394. doi:10.1091/mbc.E16-01-0063

11) Fig. 3D: why are these effects not tested in an ANOVA that takes all mutants and wild-type into consideration, rather than pair-wise t-tests? That would be better practice.

We are not sure what the reviewer means here. All statistical tests in Fig 3 were ANOVA tests, no pairwise t-tests. No further action was taken.

**Reviewer #2:** This is a very interesting paper analysing the dynamics of two different types of MGEs, the Salmonella genomic island 1 (SGI1) and the IncC plasmids. Interestingly, while SGI1 requires IncC plasmids for mobility, these two elements are incompatible. This manuscript analyses the genetic causes of this incompatibility. Overall, this is a nice piece of work, and I just have some minor comments that hopefully will improve the manuscript:

- In a very low percentage of cells, both elements may persist, suggesting they have mutated to avoid the incompatibility. It would be nice to sequence some of these stable elements. This could identify additional genes involved in the process.

We are currently studying this phenomenon for an upcoming manuscript.

- Are the authors completely convinced that the SGI int mutant does not replicate in situ? Maybe this could be specifically tested, using the qPCR utilized in the study for the wt plasmid, for example.

We cannot rule out that locked-in SGI1 does partially replicate in situ. Initiation of replication in this context likely leads to incomplete copies of SGI1 fused to chromosomal DNA. Although such chimeras are not representative of SGI1’s natural context and are probably irrelevant to its physiology, we modified the text of the discussion to integrate this possibility (lines 396-398).

- Is Rep (together with its cognate oriV site) sufficient for autonomous SGI1 replication? Maybe the authors could clone these two elements in a suicide plasmid to test if they can support SGI1 replication.

This a very interesting question. We have cloned *rep* and *oriV* on a Pir-dependent replicative plasmid (R6K-based). Surprisingly, we found that in cells lacking Pir, this plasmid was unable to maintain autonomously, even with AcaCD provided in trans. This suggests that another factor encoded either by SGI1 or the helper plasmid is required for stable replication. Further experiments are ongoing to determine the missing factor(s).

- I would like to see some discussion about the evolutionary advantage of this relationship, especially for the island? It is nice but strange that one parasitic element, the island, which requires the plasmid for transfer, can’t cohabit with the helper plasmid.

We thank the reviewer for this remark. We have now added a paragraph regarding the evolutionary advantage of this relationship for SGI1 in the discussion (lines 490-506).

**Reviewer #3:** These authors present a careful and clever analysis of co-existence of conjugative plasmids and ICE-like elements in Salmonella. Overall, I thought the paper makes a nice contribution to the field and is well written (albeit maybe a little wordy), with nice illustrations and figure design. I have no major problems with the ms as presented here, and my comments refer to potential follow up studies that might give a little more impact to this paper. They are as follows:

We appreciate the thoughtful comments of this reviewer and his/her suggestions of experiments for future studies.

1) In the primary experiment shown in Fig. 2, the authors were able to select out rare clones (undetectable by flow cytometry) where both elements were still co-resident-- it would be interesting to plate these on antibiotic plates, select some colonies (maybe 10 or so) and repeat the passage in the absence of selection to see if they get the same result-- it is possible that there could have been selections for suppressor mutants of the replication/incompatibility functions encoded by the wild type elements and these might further inform the mechanisms.

We are currently studying this phenomenon and suspect the emergence of mutants of SGI1 unable to excise or replicate, or mutants of the helper plasmid that are unable to produce a function AcaCD activator complex.

2) The authors might have published this previously, but it would be interesting t know whether there is a basal level of transfer (eg. solid surface matings) of the Island in the absence of a helper plasmid.

Although SGI1 encodes 3 T4SS subunit, it lacks most of the genes required for the assembly of a complete and functional T4SS. Hence, SGI1 is unable to transfer in the absence of an helper IncA or IncC plasmid. This has been previously shown by several authors in different independent studies. See Douard G, Praud K, Cloeckaert A, Doublet B. The *Salmonella* genomic island 1 is specifically mobilized in trans by the IncA/C multidrug resistance plasmid family. PloS One. 2010;5: e15302. doi:10.1371/journal.pone.0015302 or	Carraro N, Durand R, Rivard N, Anquetil C, Barrette C, Humbert M, et al. *Salmonella* genomic island 1 (SGI1) reshapes the mating apparatus of IncC conjugative plasmids to promote self-propagation. PLOS Genet. 2017;13: e1006705. doi:10.1371/journal.pgen.1006705

Otherwise I thought this was a nice paper.
